# Engineering a pH‐responsive polymeric micelle co‐loaded with paclitaxel and triptolide for breast cancer therapy

**DOI:** 10.1111/cpr.13603

**Published:** 2024-01-16

**Authors:** Mengmeng Zhang, Na Ying, Jie Chen, Liwen Wu, Huajie Liu, Shihua Luo, Dongdong Zeng

**Affiliations:** ^1^ Shanghai University of Medicine & Health Sciences Shanghai China; ^2^ Shanghai University of Traditional Chinese Medicine Shanghai China; ^3^ Tongji University Shanghai China; ^4^ Department of Traumatology, Rui Jin Hospital, School of Medicine Shanghai Jiao Tong University Shanghai China

## Abstract

Breast cancer has overtaken lung cancer as the number one cancer worldwide. Paclitaxel (PTX) is a widely used first‐line anti‐cancer drug, but it is not very effective in clinical breast cancer therapy. It has been reported that triptolide (TPL) can enhance the anticancer effect of paclitaxel, and better synergistic therapeutic effects are seen with concomitant administration of PTX and TPL. In this study, we developed pH‐responsive polymeric micelles for co‐delivery of PTX and TPL, which disassembling in acidic tumour microenvironments to target drug release and effectively kill breast cancer cells. Firstly, we synthesized amphiphilic copolymer mPEG_2000_‐PBAE through Michael addition reaction, confirmed by various characterizations. Polymer micelles loaded with TPL and PTX (TPL/PTX‐PMs) were prepared by the thin film dispersion method. The average particle size of TPL/PTX‐PMs was 97.29 ± 1.63 nm, with PDI of 0.237 ± 0.003 and Zeta potential of 9.57 ± 0.80 mV, LC% was 6.19 ± 0.21%, EE% was 88.67 ± 3.06%. Carrier material biocompatibility and loaded micelle cytotoxicity were assessed using the CCK‐8 method, demonstrating excellent biocompatibility. Under the same drug concentration, TPL/PTX‐PMs were the most toxic to tumour cells and had the strongest proliferation inhibitory effect. Cellular uptake assays revealed that TPL/PTX‐PMs significantly increased intracellular drug concentration and enhanced antitumor activity. Overall, pH‐responsive micellar co‐delivery of TPL and PTX is a promising approach for breast cancer therapy.

## Graphical abstract

Self‐assembly of TPL/PTX‐PPMs forpH‐responsive drug release in tumor cells.

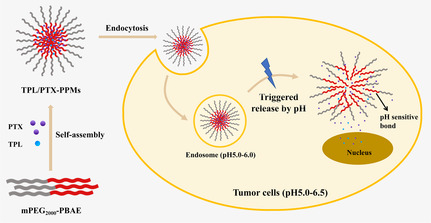

## 1 INTRODUCTION

Breast cancer added 2.26 million people worldwide in 2020, overtaking lung cancer (2.21 million) as the number one cancer worldwide.^1^ Traditional cancer treatments include surgery, radiotherapy, chemotherapy, and immunotherapy.^2^ Drug therapy is an integral part of the long‐term treatment of almost all breast cancer patients.^3^ Paclitaxel (PTX), a first‐line treatment drug for breast cancer, is a microtubule stabilizer that accelerates microtubule polymerization and stabilization, disrupts cell mitosis, and ultimately leads to apoptosis.^4^, ^5^, ^6^, ^7^ Due to the limitations of drug resistance, the effectiveness of PTX in treating breast cancer does not meet expectations.^8^, ^9^ Triptolide (TPL) has potent antitumor activity, which exerts anticancer effects by affecting a variety of molecules and signalling pathways, such as heat shock proteins, caspase, NF‐κB and DNA repair‐related factors.^10^, ^11^, ^12^ The combination of TPL and PTX can improve the anti‐tumour effect of PTX.^13^ The antitumor effect of TPL combined with PTX is mediated by induction of apoptosis, and TPL enhances PTX‐mediated apoptosis by suppressing NF‐kB activity.^13^ In addition, combination chemotherapy is effective in reducing drug dosage and toxicity, and can also reverse multidrug resistance.^14^, ^15^

However, the different water solubility of PTX and TPL limits their combined use in clinical settings.^16^ Therefore, finding a suitable drug delivery system to deliver PTX and TPL becomes crucial for combination therapy.

In recent years, the rise of nanotechnology has facilitated the development of nanodrug delivery systems (NDDS). NDDS provides a promising method for controlled and targeted drug delivery due to its good biocompatibility,^17^ low side effects,^18^ targeting,^19^ and controlled release,^20^ and is one of the prospective strategies for cancer therapy.^21^, ^22^, ^23^ NDDS include nanoparticles, liposomes, polymeric micelles, polymeric drug couplers, etc. Polymeric micelles (PMs), a self‐assembled carrier of amphiphilic copolymers, are widely used in anticancer drug delivery systems because of their low toxicity, long duration in the blood circulation, and evasion of drug resistance.^24^, ^25^, ^26^, ^27^ PMs are a thermodynamically stable colloidal solution formed by the self‐assembly of synthetic amphiphilic block copolymers in water through various driving forces such as hydrophobic and electrostatic interactions. It has a size of 10–100 nm and a unique core–shell structure.^28^ Its small size makes it difficult to be recognized by the liver and reticuloendothelial system, thus avoiding rapid clearance of anti‐cancer drugs by the kidneys, prolonging the circulation time of drugs in the blood, and maintaining the biological activity and function of anti‐cancer drugs in the body.^29^, ^30^, ^31^ Most anti‐cancer drugs are hydrophobic in nature.^32^ The special core–shell structure of PMs has a hydrophobic core with a hydrophilic coating, so that hydrophobic drugs can be encapsulated in the hydrophobic core of PMs.^33^ Moreover, the modification of its hydrophilic segments to give it a specific response can control the release of the drug from PMs at the tumour site, thus reducing the adverse effects caused by the drug.^34^, ^35^ PMs can also passively target drugs to solid tumours through the enhanced permeability and retention (EPR) effect to reduce the toxic side effects of anticancer drugs, solving the problem of untargeted conventional chemotherapy drugs.^36^, ^37^ Herein, we have prepared a PTX and TPL co‐delivery polymeric micelle (TPL/PTX‐PPM) using pH‐responsive amphiphilic block copolymer carrier materials, which is capable of co‐releasing PTX and TPL in the acidic microenvironment of tumours. Moreover, its size suggesting an EPR effect, enabling TPL/PTX‐PPM a desirable anticancer effect, thus providing an effective approach to the treatment of breast cancer.

## 2 MATERIALS AND METHODS

### 2.1 Chemicals and reagents

Polyethylene glycol (PEG, Mn2000), Trichloromethane, Acrylylchloride, Triethylamine, Dichloromethane, 1,6‐Hexanedioldiacrylate, 1,3‐Bis(4‐piperidyl) propane, Ethyl ether, Methanol and Acetonitrile were purchased from Shanghai Titan Scientific Co., Ltd. (China). Paclitaxel (≥99.0%) and Triptolide (≥98.0%) were obtained from Shanghai Macklin Biochemical Co., Ltd. (China). Trypsin solution, DMEM medium, Penicillin–streptomycin solution and Serum‐Free Cell Freezing Medium were purchased from Sangon Biotech, China.

### 2.2 Cell culture

Human breast cancer cells (MDA‐MB‐231 cells) were purchased from Sangon Biotech, China. MDA‐MB‐231 cells were cultured in a complete medium (DMEM medium, high glucose + 10% Fetal bovine serum + 1% Penicillin‐streptomycin solution). All cells were cultured at 37°C in a 5% CO_2_ atmosphere.

### 2.3 Synthesis of amphiphilic copolymers (mPEG_2000_‐PBAE)

**SCHEME 1:**
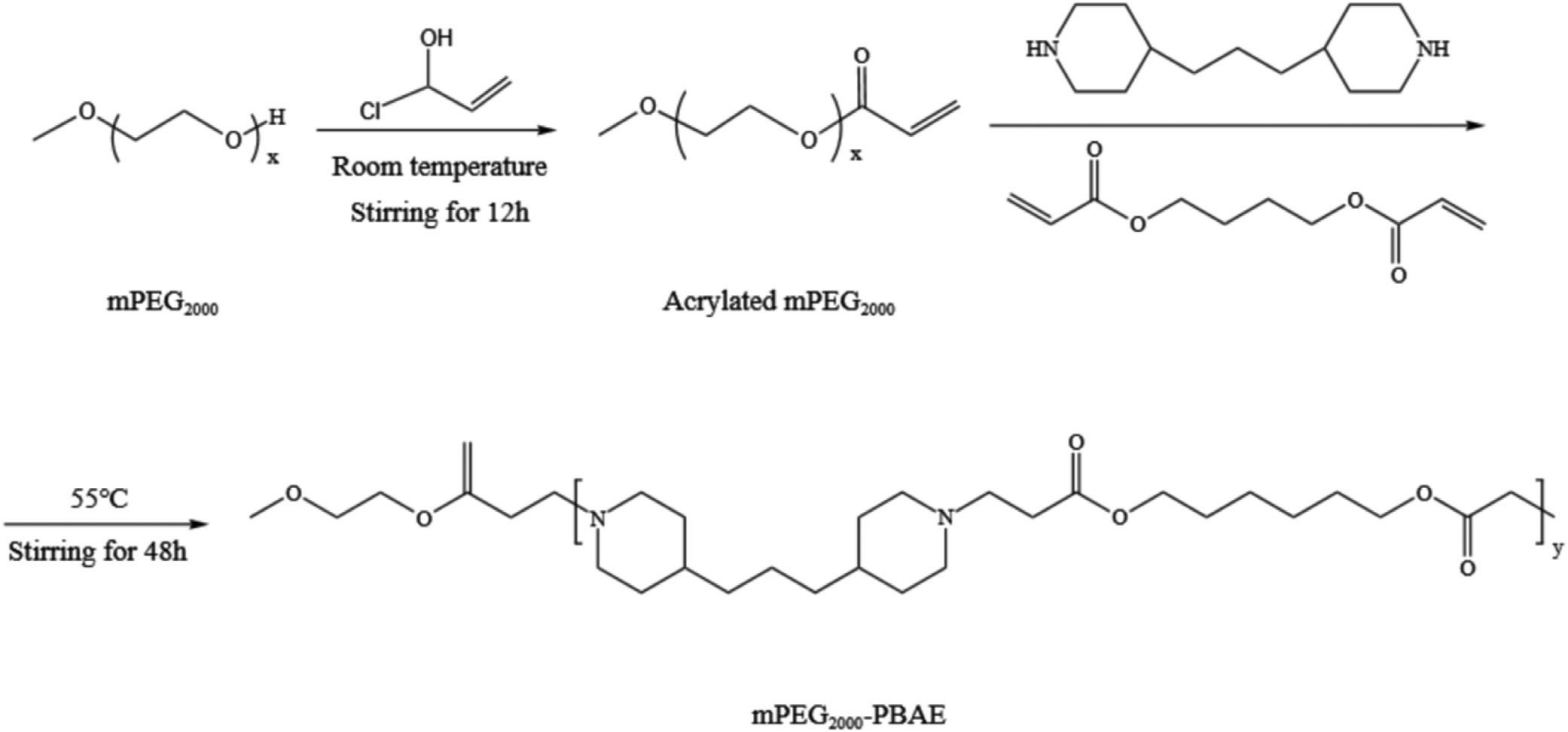
Synthetic route of acrylated mPEG_2000_ and mPEG_2000_‐PBAE.

The acrylate mPEG_2000_ and mPEG_2000_‐PBAE amphiphilic copolymer were synthesized by Michael addition reaction (Scheme 1). mPEG_2000_ was dissolved in 20 mL of trichloromethane (maintain reaction temperature at 4°C). Then, acryloyl chloride (2.0 eq.) and triethylamine (3.0 eq.) were added slowly dropwise until dissolution was complete. The reactant was stirred at room temperature for 12 h. After the reaction, the reactant was washed and dried, then concentrated under reduced pressure and poured into the cold ether, and the precipitation was collected by centrifugation. The above precipitate was dried under vacuum for 12 h to give a white acrylate mPEG_2000_.

Acrylate mPEG_2000_ was dissolved in 20 mL of dichloromethane, then 1,6‐Hexanedioldiacrylate (10.0 eq.) and 1,3‐Bis(4‐piperidyl) propane (11.0 eq.) were added and completely dissolved. The reaction was stirred at 55°C for 48 h under nitrogen. The reaction solution was concentrated under reduced pressure and poured into a large amount of cold ether to precipitate it. The product was collected by centrifugation. The mPEG_2000_‐PBAE amphiphilic copolymer was obtained by vacuum drying and stored at −20°C for backup.

### 2.4 Characterization of mPEG_2000_‐PBAE

mPEG_2000_‐PBAE was characterized by Fourier transform‐infrared spectroscopy (FT‐IR, Spectrum Two DTGS, PerkinElmer Llantrisant, England) and nuclear magnetic resonance (^1^HNMR, 400 MHz, JNM‐ECZ400S/L1, JEOL, Japan) in CDCl_3_.

The critical micelle concentration (CMC) of mPEG_2000_‐PBAE was determined by fluorescence spectroscopy (Hitachi, Japan) using pyrene as a hydrophobic probe. First, 1 mL of 5 × 10^−6^ mol/L pyrene acetone solution was placed in a series of sample bottles and the acetone was made to evaporate by nitrogen gas. Then 2 mL of copolymer solution with a concentration gradient of 1 × 10^−4^ to 1 mg/mL was added to the sample bottle containing pyrene and ultrasonicated in a water bath at 55°C for 2 h. All samples were then left at room temperature overnight. The fluorescence intensity of pyrene in the above series of polymer solutions was scanned in the range of 300–360 nm, and the fluorescence intensities (I336 and I338) at 336 nm and 338 nm were recorded for each concentration of copolymer. The CMC of polymer mPEG_2000_‐PBAE was fitted using Origin2018 software.

### 2.5 Preparation of mPEG_2000_‐PBAE micelles

#### 2.5.1 Screening of the co‐loading ratio of TPL and PTX

MDA‐MB‐231 breast cancer cells were used as model cells, and the proliferation inhibition rate of TPL and PTX alone or in combination in different ratios (TPL:PTX = 1:2, 1:4 and 1:6) on MDA‐MB‐231 cells was detected by Cell Counting Kit‐8 (CCK‐8) method. The TPL concentrations of 2.5, 5, 10, 20, 40 and 80 μg/mL were fixed for each group of mixture. And the median‐effect equation and drug combination index (CI) were used to evaluate the drug combination effect and screen out the optimal combination ratio of TPL and PTX for MDA‐MB‐231 cells. The median‐effect equation (Equation 1) is commonly used to evaluate the effect of drug combinations.^38^

(1) $$\log\left(\frac{\mathrm{fa}}{\mathrm{fu}}\right)=m\log\left(D\right)-m\log\left(\mathrm{Dm}\right)$$

If *b* = *m*, *a* = −*m*log (Dm), *Y* = log (fa/fu), *X* = log (*D*), then *Y* = *bX* + *a*.

*D* is the dose (or concentration) of the drug, fa is the fraction affected by *D* (i.e., percent inhibition/100), and fu is the fraction unaffected (i.e., fu = 1 − fa). Dm is the IC_50_ of MDA‐MB‐231 cells. *m* is the coefficient indicating the shape of the dose‐effect relationship, where *m* = 1 for hyperbolic curves, m>1 for S‐shaped curves, and m<1 for flat S‐shaped volume effect curves.

#### 2.5.2 Preparation of PMs and TPL/PTX‐PMs

Preparation of mPEG_2000_‐PBAE micelles with TPL/PTX (TPL/PTX‐PMs) by thin film dispersion method. First, TPL, PTX and mPEG_2000_‐PBAE dissolved together in 2 mL of methanol and ultrasound in an ice bath for 30 min. Then, the solvent was removed with a rotary evaporator to obtain a homogeneous film. Next, 10 mL of deionized water was added and hydrated under a water bath at 50°C for 0.5 h to make the thin film layer uniformly dispersed in water. Finally, the solution was filtered through 0.22 μm Millipore filter and PTX/TPL‐PMs was obtained.

The preparation of blank micelles (PMs) is the same as the preparation of TPL/PTX‐PMs. mPEG_2000_‐PBAE were dissolved in 2 mL of methanol in the first step, and other steps were unchanged.

### 2.6 Characterization of PMs and TPL/PTX‐PMs

#### 2.6.1 Stability and pH sensitivity of PMs

Particle sizes and polydispersity index (PDI) of PMs stored at room temperature for 0, 2, 4, 6, 8, 10, 12 and 14 days were determined using a particle analyser (Zetasizer Lab, Malvern, UK) to check the storage stability of PMs. The average particle size and PDI were determined by placing the PMs into PBS buffer of different pH values (in the range of 4–9) for 30 min to examine the pH sensitivity of the PMs.

#### 2.6.2 Biocompatibility of PMs in different PH environments

Biocompatibility of carrier materials was assessed by the CCK‐8 method. MDA‐MB‐231 cells was seeded on 96‐well plates at a density of 5000 cells per well. After incubation in 5% CO_2_ atmosphere at 37°C for 24 h, the old medium was removed from the well plates. The cells were then co‐cultured with different concentrations (15.625, 31.25, 62.5, 125, 250, 500 μg/mL) of PMs at the medium of different pH (pH 7.4 and pH 6.5) for 48 h. The old medium containing the carrier material was removed and 100 μL of DMEM medium containing 10% CCK‐8 solution was added to each well and placed in the incubator for 1 h. The absorbance (OD) in each well was measured at 450 nm on a microplate reader. Cellular activity was calculated using Equation (2).

(2) $$\text{Cell viability}\left(\%\right)=\left[\frac{\mathrm{As}-\mathrm{Ab}}{\mathrm{Ac}-\mathrm{Ab}}\right]\times 100$$

As = absorbance of the experimental well (absorbance of cells, medium, CCK8 and wells of the test compound); Ab = blank well absorbance (absorbance of wells containing medium and CCK8); Ac = control well absorbance (absorbance of wells containing cells, medium and CCK8).

#### 2.6.3 Particle size, zeta potential and morphology of TPL/PTX‐PMs

TPL/PTX‐PMs were diluted with ultrapure water and the particle size and zeta potential of TPL/PTX‐PMs were determined by a particle analyser (Zetasizer Lab, Malvern, UK). The surface morphology of TPL/PTX‐PMs were analysed by scanning electron microscopy (Hitachi, Japan). The samples were analysed for morphology using a field emission scanning electron microscope (Regulus 8100, HITACHI, Japan) with a voltage of 10 KV.

#### 2.6.4 Drug loading and encapsulation efficiency of TPL/PTX‐PMs

The masses of PTX and TPL were determined at different wavelengths using high performance liquid chromatography (HPLC) (Agilent 1260 Infinity, Agilent, USA). The drug loading (DL) and encapsulating efficiency (EE) were calculated by Equations (3) and (4).

(3) $$\mathrm{EE}\%=\frac{\text{Total drug}-\text{Unentrapped drug}}{\text{Total drug}}\times 100\%$$

(4) $$\mathrm{DL}\%=\frac{\text{Total drug in PPMs}}{\text{Total weight of PPMs}}\times 100\%$$

#### 2.6.5 Storage stability of TPL/PTX‐PMs

The mean particle size and DL% of TPL/PTX‐PMs were used as indicators to investigate the formulation stability of TPL/PTX‐PMs stored at room temperature. Samples were taken on 0, 2, 4, 6, 8, 10, 12, and 14 days for measurement, respectively.

### 2.7 pH‐responsive release of TPL/PTX‐PMs

The in vitro release of TPL/PTX‐PMs under different pH conditions was studied by dialysis. A dialysis bag (Mw 3500) containing TPL/PTX‐PMs was placed in 30 mL fresh PBS (pH 5.5, 6.5, or 7.4 containing 0.5% Tween 80(W/V)), and shaken at a speed of 180 rpm at 37°C. At predetermined times (0.5, 1, 2, 4, 6, 8, 12, 24, 48, 72, 96 h), 1.0 mL of the release solution was extracted while fresh PBS of the same pH was added to keep the volume constant. The amount of drug released was determined by HPLC and the cumulative release amounts of TPL and PTX were calculated separately according to Equation (5).

(5) $$E_{r}=\frac{V_{e}\sum_{1}^{n-1}C_{i}+V_{0}C_{n}}{m_{\text{drug}}}\times 100\%$$

$E_{r}$: cumulative drug release; *V*_*e*_: replacement volume of PBS; *C*_*i*_: drug concentration released during the *i*th replacement sampling; *V*_0_: total volume of released solution; *C*_*n*_: drug concentration released during the *n*th replacement sampling; *m*_drug_: the content of PTX or TPL in drug‐loaded micelle.

### 2.8 Cellular uptake

Coumarin 6 (C6) is a lipid‐soluble fluorescent dye that is commonly used as a hydrophobic fluorescent probe encapsulated in a nanocarrier for studies such as cellular uptake or in vivo tracing. C6‐PMs were synthesized by loading C6 into PMs according to the above TPL/PTX‐PMs preparation method. 1 × 10^4^ MDA‐MB‐231 cells were inoculated into a 35 nm diameter confocal dish and incubated for 24 h. After washing in three times with pre‐warmed PBS, free coumarin 6 (Free‐C6) and C6‐PMs were added and incubated at 37°C for 0.5 h, 2 h, and 6 h. At the end of incubation, remove the old medium and wash three times with cold PBS. The cells were fixed with 4% paraformaldehyde at room temperature and allowed to rest for 15 min. Paraformaldehyde was discarded and washed three times with cold PBS, and then the appropriate amount of DAPI staining solution was added to protect the cells from light for 10 min. After washing three times with cold PBS, 1 mL of PBS was added to observe and photograph under a confocal laser scanning microscope. The fluorescence intensity was analysed by the Image J software.

### 2.9 In vitro cytotoxicity

The cytotoxicity of TPL/PTX‐PMs was assessed by the CCK‐8 assay. The concentrations of TPL/PTX‐PMs were set at 1.25, 2.5, 5, 10, 20 and 40 μg/mL and incubated at 37°C with 5% CO_2_. The rest of the operations are consistent with biocompatibility experiments.

## 3 RESULTS AND DISCUSSION

### 3.1 Characterizations of mPEG_2000_‐PBAE

**FIGURE 1:**
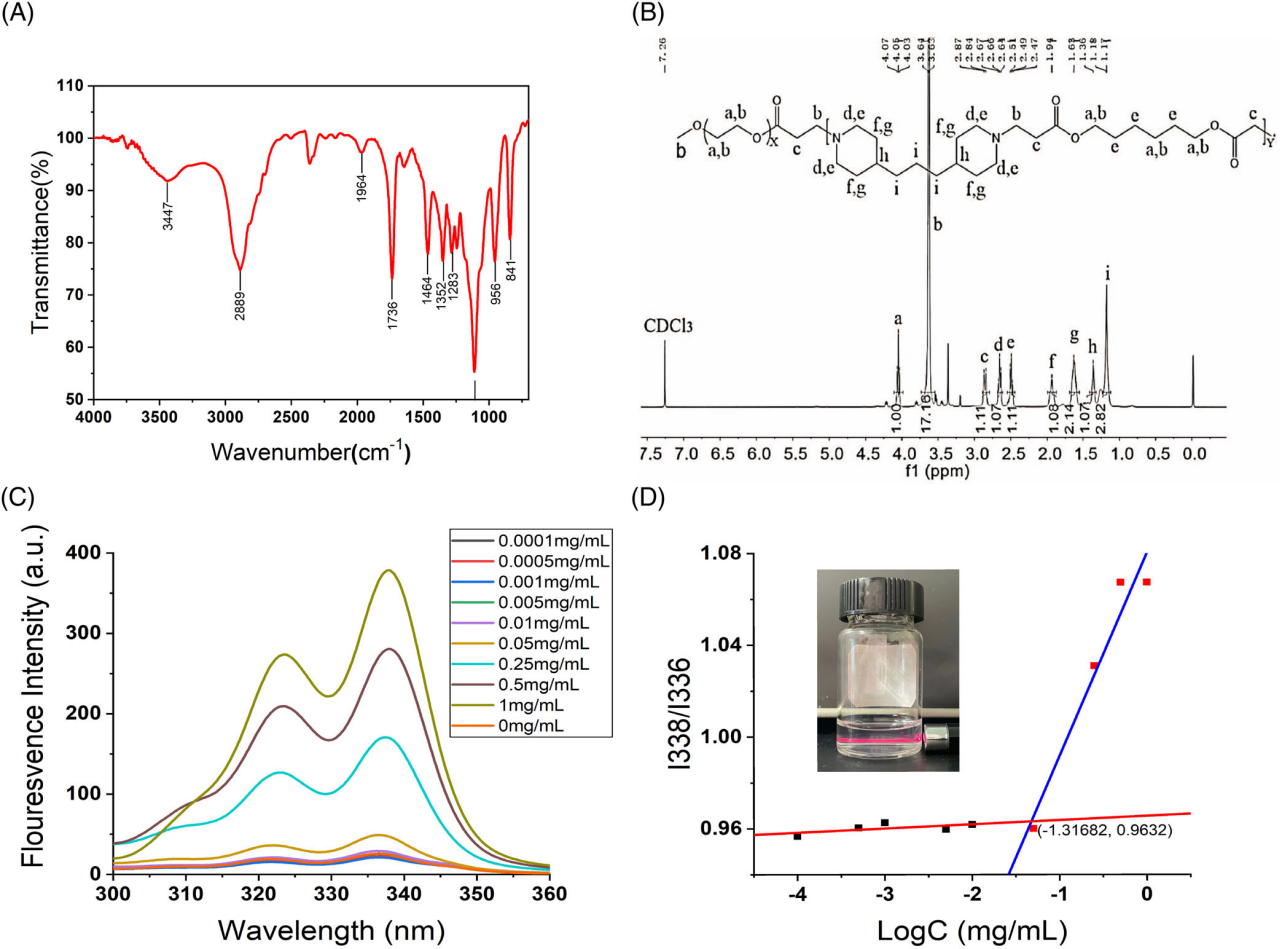
Characterization of mPEG2000‐PBAE (A) IR spectra and (B) NMR. (C) Emission spectra of pyrene as a function of copolymer concentration and (D) fluorescence intensity ratio of I338/I336 ratio from emission spectra vs. log concentration of the copolymers (the inset shows the Tyndall effect of PMs under laser irradiation).

In the FT‐IR spectrum (Figure 1A), the peak at 2889 cm^−1^ is the stretching vibration of —CH_2_— and the peak at 1244 cm^−1^ is the bending vibration of —CH_2_—. The peak located at 1736 cm^−1^ is the carbonyl absorption peak. The disappearance of the characteristic peak of the stretching vibration of the double bond (—C=C—) at 1718 cm^−1^ indicates that the double bond was broken and the mPEG_2000_‐PBAE copolymer was successfully prepared. The structure of mPEG_2000_‐PBAE was determined by ^1^HNMR spectroscopy (Figure 1B) in CDCl_3_. The chemical shift signals 2.86 ppm, 2.66 ppm, 1.94 ppm, and 1.63 ppm represent the chemical shifts of the hydrogen protons on the piperidine ring in mPEG_2000_‐PBAE, respectively. 4.05 ppm, 3.63 ppm and 2.86 ppm denote the chemical shifts of hydrogen protons in —O—CH_2_CH_2_—O—C=O—CH_2_—, respectively.

In Figure 1C, when the concentration of mPEG_2000_‐PBAE was in the range of 1 × 10^−4^–5 × 10^−2^ mg/mL, the I338/I336 ratio remained basically constant. When the concentration was greater than 5 × 10^−2^ mg/mL the I338/I336 ratio gradually decreased with increasing concentration. In Figure 1D, the intersection of the two lines is the CMC of mPEG_2000_‐PBAE, and the CMC of mPEG_2000_‐PBAE was calculated to be 4.83 × 10^−2^ mg/mL. The low CMC of the copolymer suggests that mPEG_2000_‐PBAE has the ability to self‐assemble in water and still form micellar solutions at small concentrations, facilitating the loading and delivery of lipid‐soluble drugs.

### 3.2 Preparation of TPL/PTX‐PMs

**FIGURE 2:**
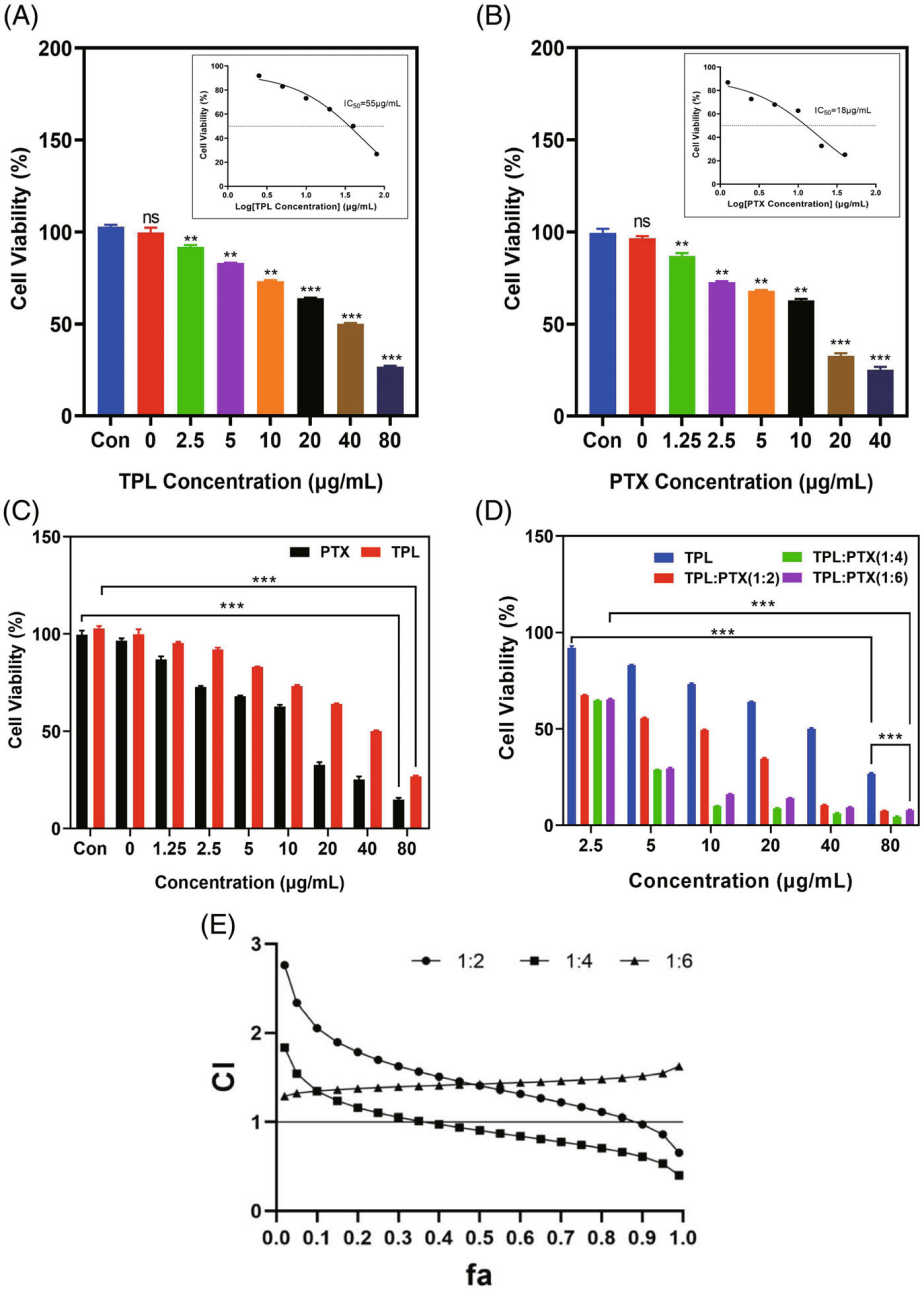
Cell viability of MDA‐MB‐231 cells after coculture with drugs for 24 h; (A) PTX; (B) TPL; (C) PTX and TPL, respectively; (D) different ratios of PTX and TPL; (E) CI curves of different ratios of TPL and PTX combined on MDA‐MB‐231 cells at fa (means ± SD, *n* = 3, ***p < 0.001).

Previous studies have shown that TPL and PTX synergize against tumours, but the ratio of TPL and PTX in combination is uncertain.^16^, ^39^ Therefore, we used MDA‐MB‐231 cells as a model to examine the ratio of the two drugs in combination. First, the inhibitory effects of TPL and PTX on the proliferation of MDA‐MB‐231 cells were examined by CCK8 assay, respectively. The results showed that both TPL and PTX had significant growth inhibitory effects on MDA‐MB‐231 cells, and the degree of inhibition showed a positive correlation with the single drug concentration (Figure 2A,B). Moreover, it is clear from Figure 2C that the minimum effect concentration of TPL is approximately twice that of PTX. TPL at 1.25 μg/mL could inhibit cell growth by 10%–20%, while PTX required 2.5–5 μg/mL to achieve the same inhibitory effect. It further indicates that TPL has a stronger inhibitory effect on MDA‐MB‐231 cells compared to PTX. Based on the above results, the ratio of 1:2, 1:4, and 1:6 combination dosing of TPL and PTX were examined. In Figure 2D, it is shown that each group of the combination drug showed higher inhibition of MDA‐MB‐231 breast cancer cells than TPL alone, and the degree of inhibition was also correlated with the concentration of the combination drug group. The best inhibition of proliferation of MDA‐MB‐231 cells was achieved when the ratio of TPL to PTX was 1:4, and the inhibition of cell proliferation increased with the increase of drug concentration in the drug‐loaded micelles (*p* < 0.001). It can be indicated that the combination of the two drugs could better inhibit the proliferation of tumour cells.

Based on the above experimental results of CCK‐8, the IC_50_ of single drug was fitted using GraphPad Prism 8.0, and the IC_50_ of different ratios of combined drugs was fitted using Calcusyn statistical. Based on the median‐effect equation, regression equations and correlation coefficients (*r*) were obtained for the single‐drug group and the group administered in different ratios in combination (Table S1). It can be seen that the inhibitory effect of PTX alone on MDA‐MB‐231 cells is stronger than that of TPL alone. The IC_50_ for both TPL and PTX combined administration was significantly lower than that of the group using PTX alone, and the IC_50_ values decreased significantly with an increasing proportion of PTX. This indicates that PTX enhances the proliferation inhibitory effect of TPL on MDA‐MB‐231 cells. Calcusyn statistical software was applied to derive the association index (CI) when different ratios of TPL and PTX were combined to act on MDA‐MB‐231 cells (Table S2). The CI curves for the combination of TPL and PTX at different ratios are shown in Figure 2E. In the figure, CI > 1 indicates antagonism, CI = 1 indicates addition effect, and CI < 1 indicates synergistic effect. The fa was between 0.5 and 0.8 to indicate the strong synergistic effect of the drug combination. Therefore, when the combination drug ratio of TPL and PTX is 1:4, the CI curve is mostly in the high effect area (CI < 1), so the combination drug at this ratio can exert a better anti‐tumour effect. In overview, we chose to load TPL and PTX into PMs in a 1:4 ratio for subsequent studies. Specifically, the final concentration of TPL in the drug‐loaded micelles was 50 μg/mL, the final concentration of PTX in the drug‐loaded micelles was 200 μg/mL, and the total drug concentration of the micelles was 250 μg/mL.

### 3.3 Characterization of PMs and TPL/PTX‐PMs

#### 3.3.1 Stability and pH sensitivity of PMs

**FIGURE 3:**
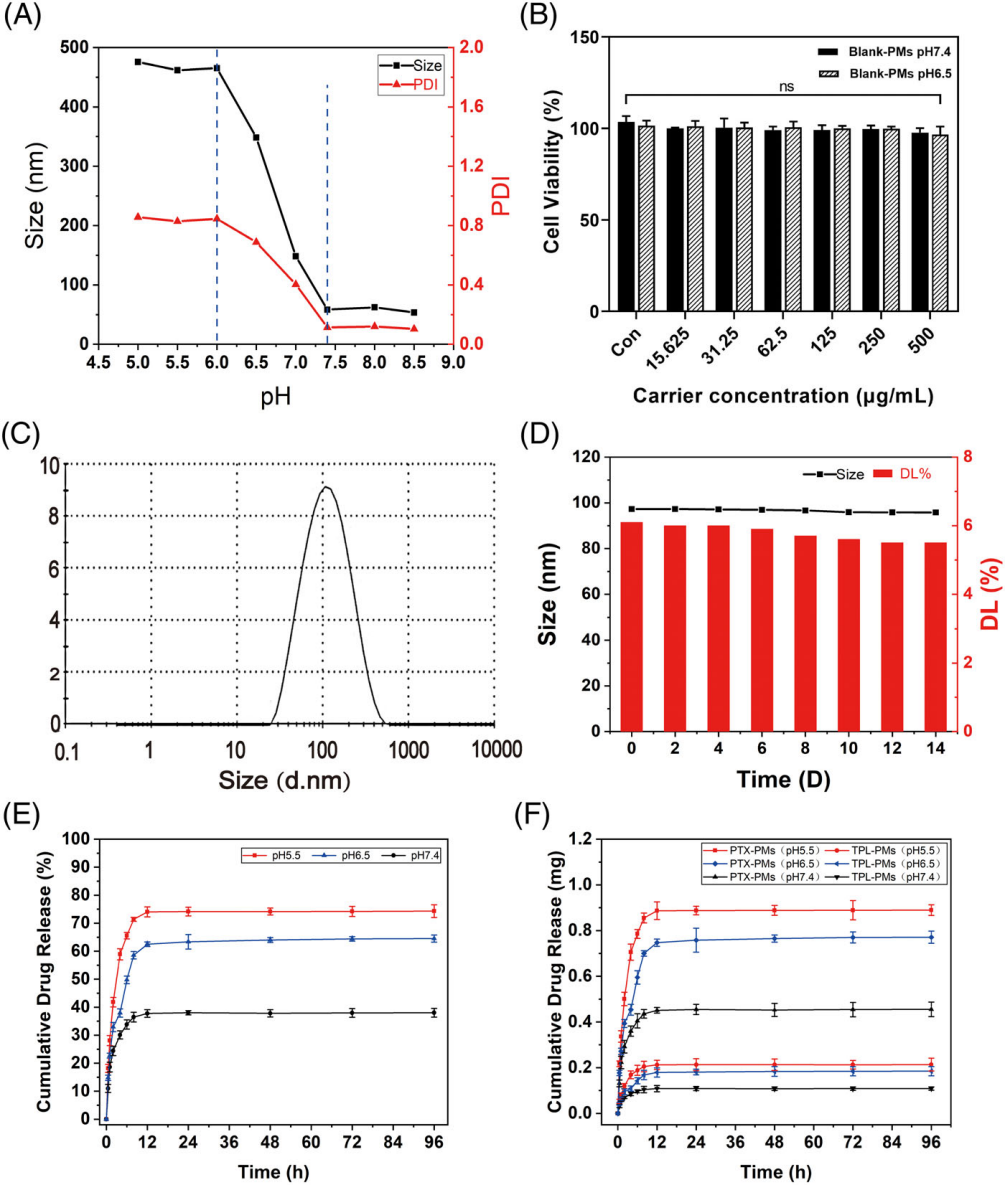
(A) Variation of particle size and PDI of PMs under different pH conditions. (B) Biocompatibility of MDA‐MB‐231 cells after co‐culture with PMs at different pH (pH 6.5, pH 7.4) for 48 h. Characterization of TPL/PTX‐PMs. (C) Particle size distribution; (D) Trends in particle size and DL% of PMs over 14 days; (E) Cumulative release rate curves of TPL and PTX at different pH; (F) Cumulative release amount curves of TPL or PTX at different pH (means ± SD, *n* = 3, ns *p* > 0.5).

Colloidal storage stability is one of the important factors to evaluate the merits of nano‐delivery systems. We investigated the stability of PMs, and the results showed that the particle size and PDI of PMs hardly changed within 2 weeks (Figure S1a), indicating good stability of PMs. The changes in the particle size of PMs at different pH conditions are shown in Figure 3A. When the pH was higher than 7.4, the change in particle size was not obvious. pH between 7.4 and 6.0 showed a significant increase in the particle size of PMs up to about 500 nm. In this pH range, the tertiary amine group in PBAE undergoes protonation, causing a gradual change in the hydrophobic segment of the amphiphilic material, which results in a swelling of the carrier structure and leads to an increase in particle size. After the pH was reduced to below 6.0, the particle size basically stopped increasing because the protonation of tertiary amine was close to saturation. The results indicate that the prepared PMs are pH sensitive.

#### 3.3.2 Biocompatibility of PMs in different PH environments

The biocompatibility of nano‐delivery carriers is one of the important factors to be considered for the future clinical application of novel drug formulations. The proliferation inhibition of MDA‐MB‐231 cells by self‐assembled micellar carrier materials was assessed by CCK‐8 assay. We tested the effect of PMs at concentrations ranging from 15.625 to 500 μg/mL on cell viability at pH 6.5 and 7.4, respectively. The results are shown in Figure 3B. Cell viability was still close to 100% after 48 h of co‐culture of MDA‐MB‐231 with PMs ranging from 15.625 to 500 μg/mL at pH 6.5 and pH 7.4. This indicates that the carrier materials self‐assembled into micelles have good biocompatibility in both pH 6.5 and pH 7.4 environments.

#### 3.3.3 Particle size, zeta potential and morphology of TPL/PTX‐PMs

The average particle size of TPL/PTX‐PMs prepared by the thin film dispersion method was 97.29 ± 1.63 nm, with a PDI of 0.237 ± 0.003 and a zeta potential of 9.57 ± 0.80 mV (Figures 3C, S1b, S1c). The micelles were observed under scanning electron microscopy, and the micelles were spherical in shape and had a three‐dimensional appearance (Figure S1d).

#### 3.3.4 Drug loading and encapsulation efficiency of TPL/PTX‐PMs

Calculated according to the formula, LC% was 6.19 ± 0.21%. EE% was 88.67 ± 3.06%. The change in the mean particle size and drug loading of TPL/PTX‐PMs over 14 days are shown in Figure 3D. The micelle particle size changed very little over the 2 weeks, while the drug loading decreased slightly from day 8 to day 14 by 4.79%. The decrease in drug loading may be due to a slight drug leakage of TPL/PTX‐PMs in the second week, corresponding to a slight decrease in micelle size from 96.67 nm to 95.74 nm.

### 3.4 pH‐responsive release of the drug

The rapid growth of the tumour results in a lack of nutrients and oxygen in some tissues, producing acid metabolites. The accumulation of these acid metabolites causes the pH of the tumour microenvironment to be slightly lower than that of normal tissues.^40^, ^41^ pH‐responsive nano‐drug delivery systems loaded with antitumor drugs can be released at an acidic pH to achieve targeted drug release in the tumour microenvironment. TPL/PTX‐PMs are pH‐responsive nano‐controlled release formulations with a core–shell structure. The results of in vitro release showed that the drug release of TPL/PTX‐PMs was pH‐dependent (Figure 3E). Under acidic (pH 5.5) conditions, TPL/PTX‐PMs undergo ionically disassembling, so that the drug is released abruptly, with 65.57% of the drug released from TPL/PTX‐PMs in the first 6 h, and the percentage of drug released reaches a maximum in the following 6–12 h. Abrupt release of TPL/PTX‐PMs was also observed under mildly acidic conditions (pH 6.5), but the maximum percentage of drug release was only 64%, which was lower than that under acidic conditions. Under neutral conditions (pH 7.4), because the structure of TPL/PTX‐PMs is basically stable, the drug can only be released through leakage, so the drug release curve showed a natural slow‐release trend. In addition, the cumulative release profile of the drug amount (Figure 3F) also showed a similar trend to the cumulative percentage release profile. Because the content of PTX in TPL/PTX‐PMs was higher than that in TPL, the release amount of PTX was also higher than that in TPL, which was consistent with the drug delivery ratio when the micelles were initially prepared. After 2, 6, 12, 24 and 48 h of TPL/PTX‐PM release in release medium at pH 5.5, the TPL: PTX release ratios were about 1:4.16, 1:4.15, 1:4.24, 1:4.24, respectively (Figure S2). This is consistent with the proportion of drugs encapsulated into micelles (TPL:PTX = 1:4). From the above results, it can be seen that TPL/PTX‐PMs has a fast pH response, and TPL and PTX burst out from TPL/PTX‐PMs under acidic or mildly acidic conditions, while under normal physiological conditions, the release rate of TPL and PTX was not high, and the cumulative release amount was also low. It shows that it has certain biological safety and will not kill normal cells.

### 3.5 Cellular uptake

Cellular uptake experiments use the green fluorescence of C6 to localize the distribution of micelles within the cell, and the nucleus is localized by DAPI (blue) staining, thus simulating the distribution of the drug being loaded after being endocytosed into the cell. The fluorescence distribution of Free‐C6 and C6‐PMs in MDA‐MB‐231 cells was observed by a confocal laser scanning microscope.

**FIGURE 4:**
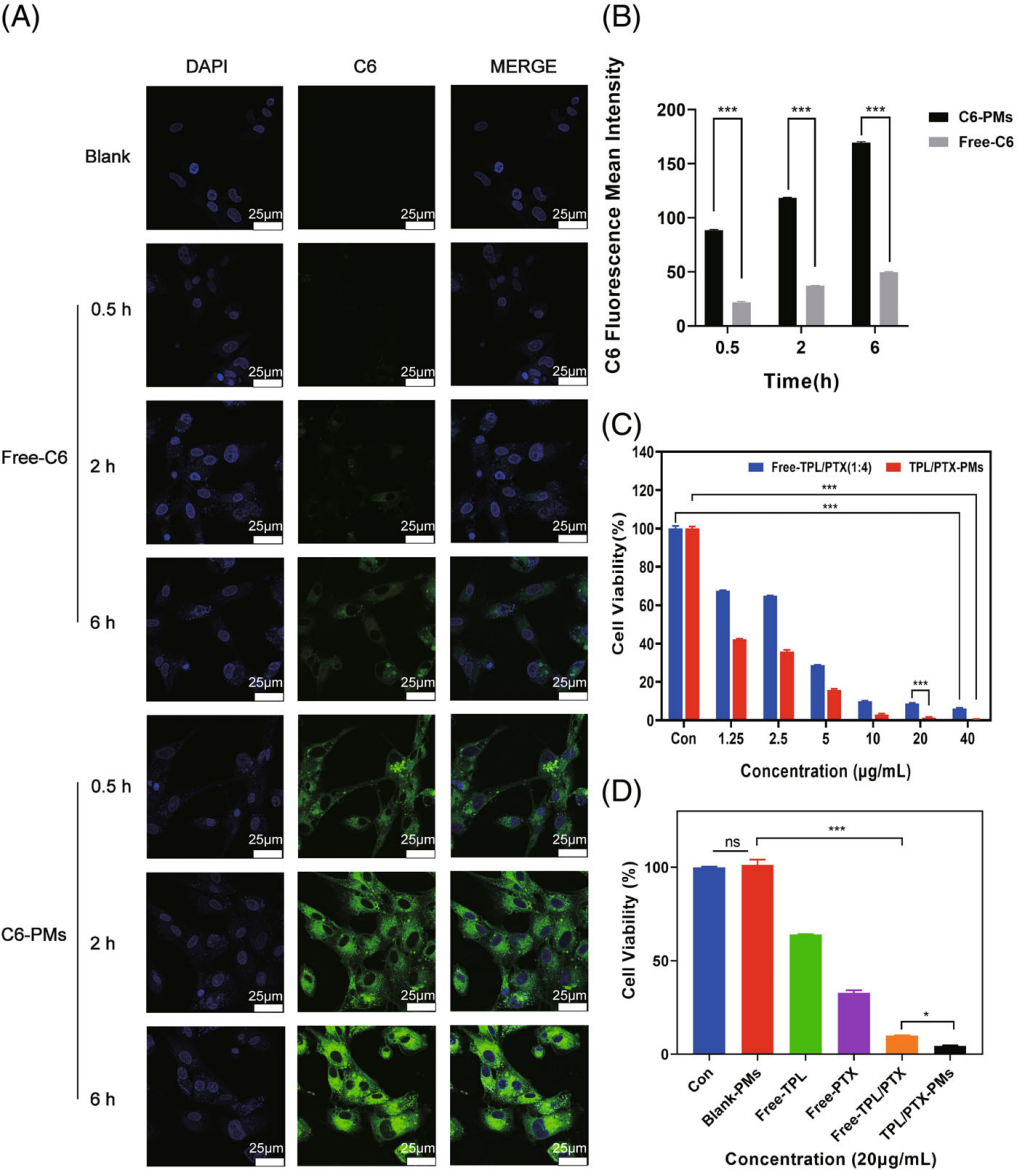
(A) Confocal laser scanning microscope observation of the uptake of Free‐C6 and C6‐PMs by MDA‐MB‐231 cell lines incubated for 0.5 h, 2 h, and 6 h, respectively. (B) Quantitative fluorescence analysis of Free‐C6 and C6‐PMs in MDA‐MB‐231 cells incubated for 0.5 h, 2 h, and 6 h, respectively. Cytotoxicity of (C) Different concentrations of TPL/PTX‐PMs and Free TPL/PTX (1:4) and (D) different treatment groups (the drug concentration in each group was 20 μg/mL) on MDA‐MB‐231 cells (means ± SD, *n* = 5, ns *p* > 0.5, *p < 0.5, ***p < 0.001).

Figure 4A shows that the green fluorescence intensity of C6‐PMs was obviously higher than that of Free‐C6 in MDA‐MB‐231 cells. The fluorescence intensity of C6‐PMs reached the maximum after 6 h of co‐ incubation, and this time coincided with the drug release time of TPL/PTX‐PMs, that is, the drug release of TPL/PTX‐PMs increased steeply from 6 h under acidic conditions. This result indicated that C6‐PMs increased the uptake of MDA‐MB‐231 cells and increased drug accumulation in tumour tissue sites.

As shown by the quantitative fluorescence analysis (Figure 4B, Table S3), the Free‐C6 fluorescence intensity at 0.5 h incubation was very low and almost not observed. At 6 h incubation time, although the Free‐C6 fluorescence intensity reached 49.7 ± 0.56, it was only half of that of C6‐PMs incubated for 0.5 h. The fluorescence intensity of C6‐PMs was always about 4 times higher than that of Free‐C6 under the same incubation time. In summary, the co‐delivery of TPL and PTX with PMs significantly enhanced the intracellular concentration of the drugs and thus will have strong antitumor activity.

### 3.6 In vitro cytotoxicity

In order to investigate the optimal dose of TPL/PTX‐PMs to inhibit the proliferation of tumour cells, we tested the inhibitory rate of TPL/PTX‐PMs on the proliferation of MDA‐MB‐231 cells in a concentration range of 1.25–40 μg/mL. As shown in Figure 4C, the proliferation inhibition rate of TPL/PTX‐PMs and Free‐TPL/PTX on MDA‐MB‐231 cells increased with the increase of drug concentration, and the anti‐tumour effect was dose‐dependent. However, we can clearly find that only 5 μg/mL of TPL/PTX‐PMs can reduce the viability of tumour cells to less than 20%, indicating that the anticancer activity of TPL/PTX‐PMs, although dose‐dependent, has a strong anticancer effect at low doses.

The cytotoxicity of PTX and TPL synergistically on MDA‐MB‐231 cells was determined by CCK‐8 assay. As shown in Figure 4D, TPL/PTX‐PMs had the greatest cytotoxicity and the strongest inhibitory proliferation effect on MDA‐MB‐231 cells. At the same dose, the cytotoxicity of TPL/PTX‐PMs was about 15 times greater than that of the free TPL, 8 times greater than that of the free PTX, and 2 times greater than that of the free TPL/PTX. Therefore, only a low dose of TPL/PTX‐PMs is needed to achieve the proliferation inhibition effect on tumour cells that can only be achieved with a high dose of free drug. It is also for this reason that TPL/PTX‐PMs can significantly reduce the dose of the drug, and thus can effectively mitigate the damage to normal cells caused by the drug itself in clinical applications.

## 4 CONCLUSION

In conclusion, we developed amphiphilic copolymer micelles mPEG2000‐PBAE‐PMs for loading PTX and TPL, and prepared TPL/PTX‐PMs, which achieved the combined delivery of PTX and TPL to anti‐tumour effectively. The in vitro release results confirmed that the TPL/PTX‐PMs were pH‐responsive and showed explosive release of the drugs in acidic environment, which facilitated the release and accumulation of the drugs in the tumour environment. The CCK‐8 assay confirmed that the carrier materials had good biocompatibility, and the TPL/PTX‐PMs were very toxic to the tumour cells, which significantly inhibited the proliferation of the tumour cells. Cellular uptake assays showed that TPL/PTX‐PMs significantly improved the cellular uptake efficiency. The use of pH‐responsive amphiphilic polymer micelles for the delivery of TPL and PTX not only overcame the problem of poor solubility of the traditional anticancer Chinese medicines PTX and TPL, but also achieved a good anticancer effect at a low dose, because of the targeted release of the drugs in the tumour cells. Therefore, TPL/PTX‐PMs have a promising future in clinical breast cancer therapy. However, anti‐tumour testing of TPL/PTX‐PMs in animal models requires further study and we will make this a research priority in our subsequent work.

## AUTHOR CONTRIBUTIONS

**Mengmeng Zhang:** Conceptualization, data curation, formal analysis, investigation, methodology, resources, software, validation. **Na Ying:** Visualization, writing—original draft, data curation, formal analysis, investigation, software, validation. **Jie Chen:** Supervision, writing—review & editing. **Huajie Liu:** Supervision, writing—review & editing. **ShiHua Luo:** Supervision, writing—review & editing. **Dongdong Zeng:** Funding acquisition, investigation, project administration, resources, validation, writing—review & editing.

## CONFLICT OF INTEREST STATEMENT

The authors declare no competing financial interest.

## Supporting information


**Data S1.** Supporting Information

## Data Availability

Research data are not shared.
