# Supplementary material for: Engineering a pH‐responsive polymeric micelle co‐loaded with paclitaxel and triptolide for breast cancer therapy
Source: Cell Prolif. 2024 Jan 16;57(6):e13603. doi: 10.1111/cpr.13603 (PMC11150136; doi:10.1111/cpr.13603)
Supplement: Supplementary file 1 — Data S1. Supporting Information [file CPR-57-e13603-s001.docx]

**Engineering a pH-responsive polymeric micelle co-loaded with paclitaxel and triptolide for breast cancer therapy**

**Mengmeng Zhang ^a, d, 1^, Na Ying ^a, d, 1^, Jie Chen^b^，Liwen Wu^a^, Huajie Liu^b,*^, Shihua Luo^c,^** ^*^**, Dongdong Zeng ^a,^** ^**^

^a^ Shanghai University of Medicine & Health Sciences, Shanghai 201318, China

^b^ Tongji University, Shanghai 200092, China

^c^ Department of Traumatology, Rui Jin Hospital, School of Medicine, Shanghai Jiao Tong University, Shanghai 200025, China

^d^ Shanghai University of Traditional Chinese Medicine, Shanghai 201203, China

**^1^ Co-first authors**

^*^ **Co-corresponding authors**

^**^ **Corresponding author at:** Shanghai University of Medicine & Health Sciences, Shanghai 201318, China

**E-mail address:** zengdd@sumhs.edu.cn (D. Zeng), jqab@163.com (S. Luo), liuhuajie@tongji.edu.cn (H. Liu)


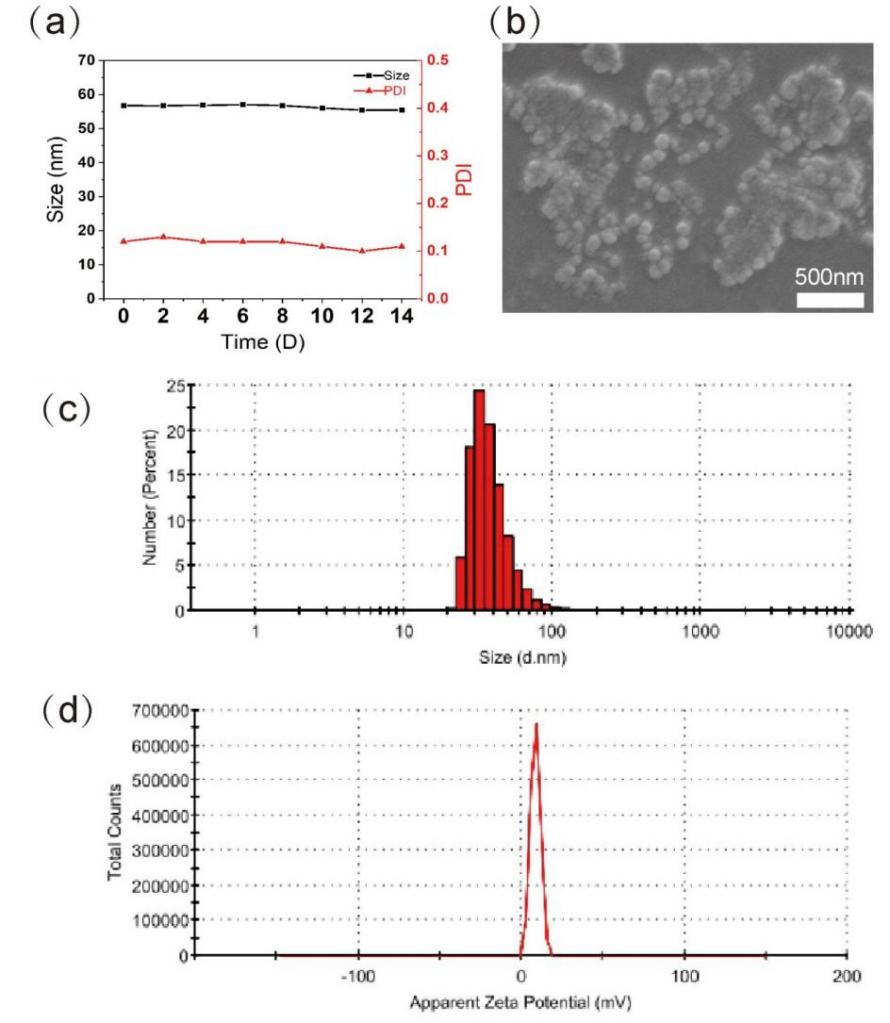


Figure S1. Characterization and biocompatibility of PPMs. (a) The particle size and PDI of PPMs in 14 days.

(b) SEM images, (c) Particle size distribution and (d) zeta potential of TPL/PTX-PPMs.

Table S1 Linear regression equations, correlation coefficients and IC_50_ values for different dosing groups

| Group | Linear regression equation | Correlation coefficient (r) | | IC_50_ |
| --- | --- | --- | --- | --- |
| TPL | Y=0.9208X-1.6025 | | 0.9919 | 55 |
| PTX | Y=0.8341X-1.047 | | 0.9733 | 18 |
| TPL: PTX (1:2) | Y=0.988X-0.8225 | | 0.9679 | 6.8 |
| TPL: PTX (1:4) | Y=0.9904X-0.3583 | | 0.9289 | 2.3 |
| TPL: PTX (1:6) | Y=0.82X-0.3263 | | 0.9284 | 2.5 |

Table S2. Combination index (CI) of different ratios of TPL and PTX combined on MDA-MB-231 cells at different affect fractions (fa).

| fa | | 0.1 | 0.2 | 0.3 | 0.4 | 0.5 | 0.6 | 0.7 | 0.8 | 0.9 |
| --- | --- | --- | --- | --- | --- | --- | --- | --- | --- | --- |
| CI | TPL: PTX  (1:2) | 2.054 | 1.785 | 1.627 | 1.509 | 1.408 | 1.315 | 1.22 | 1.114 | 0.973 |
|  | TPL: PTX  (1:4) | 1.347 | 1.162 | 1.054 | 0.973 | 0.905 | 0.841 | 0.777 | 0.705 | 0.61 |
|  | TPL: PTX  (1:6) | 1.348 | 1.376 | 1.396 | 1.412 | 1.427 | 1.443 | 1.46 | 1.482 | 1.516 |

Table S3. Results of statistical analysis of C6 uptake in MDA-MB-231 cells (n=3).

| Time（h） | | 0.5 | 2 | 6 |
| --- | --- | --- | --- | --- |
| Fluorescence mean intensity | Free-C6 | 21.8 ± 0.95 | 37.4 ± 0.13 | 49.7 ± 0.56 |
|  | C6-PPMs | 88.3 ± 0.82 | 118.3 ± 0.64 | 169.6 ± 0.74 |


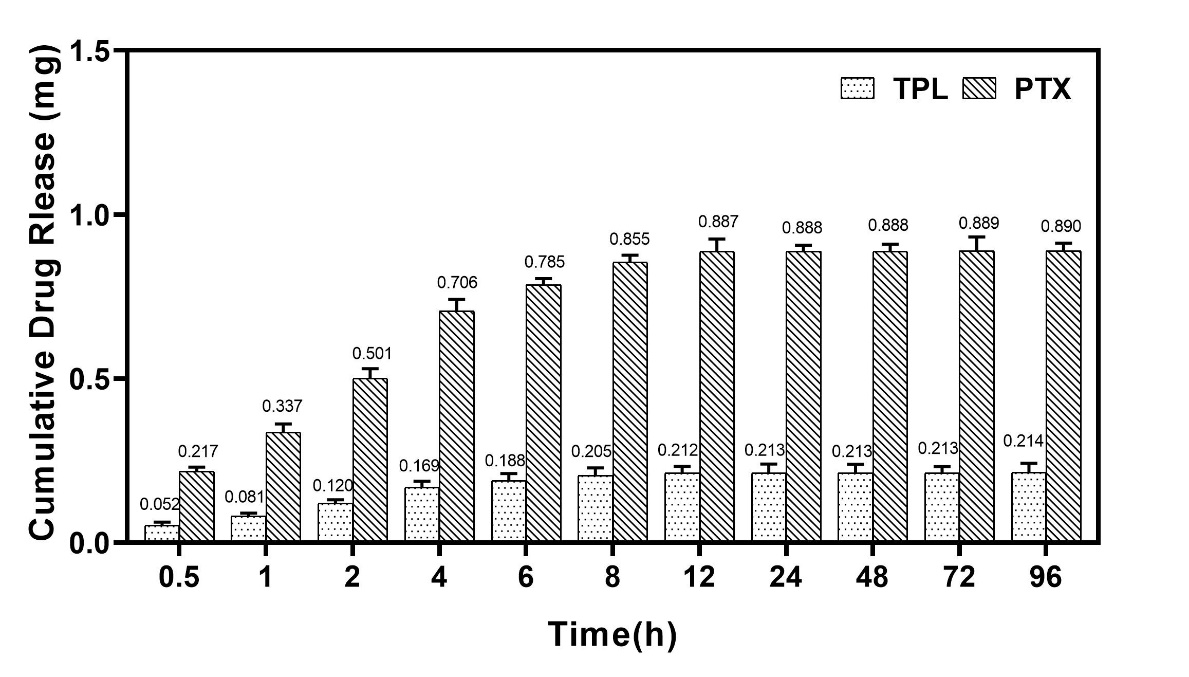


Figure S2. The ratio of drug release of TPL/PTX-PMs at different times in the release medium at pH 5.5.
